# Proposal for the Hernia ASCEND Hugo™ RAS training pathway: acquisition of skills by comprehensive exercise-based nimbleness and dexterity training

**DOI:** 10.1007/s11701-025-02704-8

**Published:** 2025-08-26

**Authors:** Francesco Brucchi, Maaike Vierstraete, Eva Vanderstraeten, Alexander Mottrie, Niki Rashidian, Filip Muysoms

**Affiliations:** 1 University of Milan, Via Festa del Perdono, 7, 20122 Milan, Italy; 2 ORSI Academy asl, Melle, Belgium; 3 AZ Heilig Hart, Lier, Belgium; 4 Department of General, HPB Surgery, and Liver Transplantation, Ghent University Hospital, Ghent, Belgium; 5 AZ Maria Middelares vzw, Ghent, Belgium

**Keywords:** Robotic-assisted surgery, Abdominal wall surgery, Proficiency-based training, Surgical training, Structured curricula, Hugo™ RAS

## Abstract

Robotic-assisted surgery (RAS) is increasingly applied in abdominal wall repair, yet structured, system-specific training remains inconsistent for emerging platforms like Hugo™ RAS. Existing literature highlights the need for dedicated curricula addressing system ergonomics, docking workflows, and procedural nuances to ensure safe adoption. The Acquisition of Skills by Comprehensive Exercise-based Nimbleness and Dexterity (ASCEND) Hugo™ RAS training pathway was developed collaboratively by ArFiCo Surgical, ORSI Academy, and Medtronic to provide a structured, modular training program for robotic hernia surgery. Building upon established da Vinci training principles, ASCEND integrates simulation, live tissue models, and mentored clinical exposure in a phased approach, ensuring progressive skill development. The pathway consists of preclinical stages, including technology training, simulator-based exercises, and high-fidelity porcine model practice for procedural steps such as robot-assisted inguinal and ventral hernia repairs. A stepwise clinical integration follows, beginning with low-complexity cases, supported by proctoring and ongoing skill assessment. Advanced modules for complex hernia techniques, including robotic transversus abdominis release (roboTAR), and dedicated “Train the Trainer” sessions, ensure scalability and standardized expertise development. The ASCEND pathway offers a comprehensive, reproducible framework for the safe, effective, and scalable adoption of Hugo™ RAS in hernia surgery. By combining Proficiency-Based Progression (PBP), simulation, and structured mentorship, ASCEND fosters surgical excellence while addressing the increasing global demand for high-quality robotic training. Further validation through multicenter studies is recommended to assess its long-term clinical impact.

**Supplementary Information:**

The online version contains supplementary material available at 10.1007/s11701-025-02704-8.

## Background

Robotic-assisted surgery (RAS) is increasingly used in abdominal wall surgery across multiple healthcare systems. Despite this growth, training programs remain non-standardized for several systems like the Hugo™ RAS platform.

Existing literature emphasizes the necessity for platform-tailored curricula to address ergonomic differences, docking workflows, and unique instrumentation handling [1]. For Hugo™ RAS, available studies are limited to simulation [2, 3], with no structured curricula published. ASCEND is the first hernia-specific, proficiency-based pathway integrating simulation, porcine models, and clinical training.

The Hernia ASCEND pathway was designed to fill this gap by addressing Hugo™-specific ergonomics, docking, and hernia-related tasks often overlooked in generic curricula. Drawing on established da Vinci protocols [4], it delivers structured and reproducible training aimed at improving surgical proficiency and ensuring patient safety.

## Methods: curriculum development

The ASCEND Hugo™ RAS Training Pathway is a stepwise program developed collaboratively by ArFiCo Surgical (Deinze, Belgium), ORSI Academy (Melle, Belgium), and Medtronic (Minneapolis, MN, USA). The pathway emphasizes progressive skill acquisition through simulation, live tissue models, and mentored clinical exposure.

Medtronic provided technical expertise regarding system specifications and logistical support during the development of the program. However, the design of the Hernia ASCEND curriculum—including its overall structure, sequencing, and definition of educational milestones—was led independently by surgical educators affiliated with ORSI Academy (Table 1).

**Table 1:**
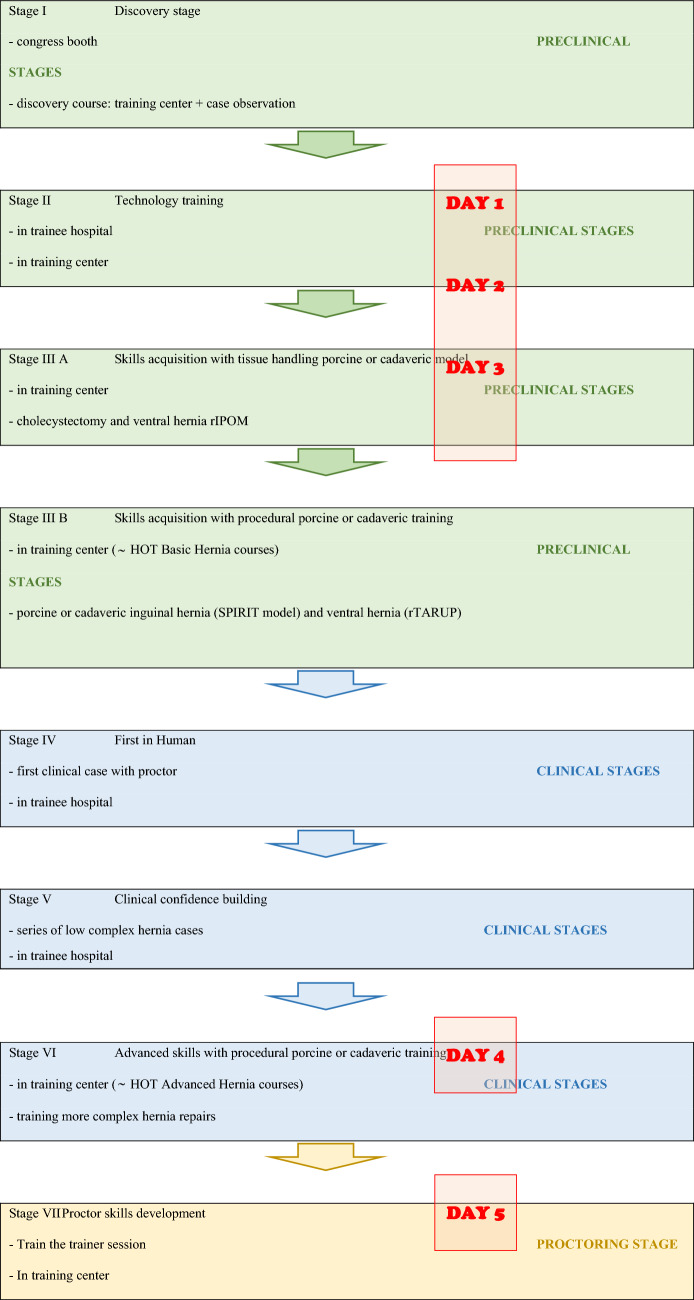
Overview of the Hernia ASCEND Hugo™ RAS training pathway

To ensure an efficient and well-organized training experience with the Hugo™ RAS robotic platform, a clear and detailed description of the pathway seems essential to allow a uniform training tailored to the need of all stake holders involved.

Drawing inspiration from the da Vinci training pathway specific for abdominal wall surgery as described by Vierstraete et al. [4], a breakdown of the pathway in phases or stages seems sensible.

## Hernia ASCEND Hugo™ RAS training pathway (Table 1)

**Fig. 1:**
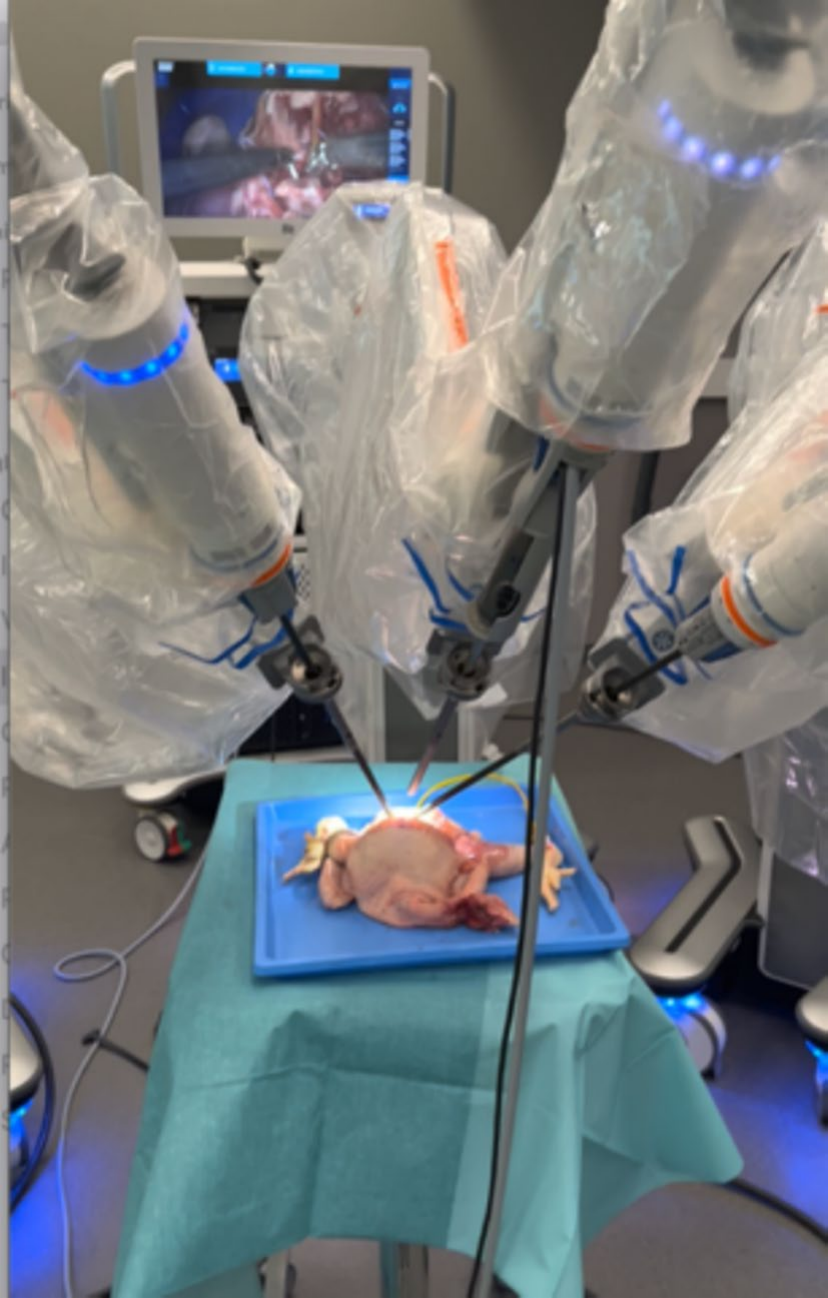
Image showing the basic skills task with chicken anastomosis and two chicken leg dissection repetitions [4], assessed using Proficiency-Based Progression (PBP) on the Hugo™ RAS system

## Stages of the Hernia ASCEND Hugo™ RAS training pathway

### Stage I—discovery stage

The primary focus of this stage is awareness-building and familiarization rather than technical skill development. Surgeons, hospital administrators, and other key stakeholders are introduced to the Hugo™ RAS robotic platform. Engagement opportunities include society congresses, where dedicated booths showcase the system, or mobile demonstrations such as the Hugo™ RAS truck.

Participants gain direct exposure to the platform’s hardware and can experience the robotic console through guided test drives. Basic simulation exercises and comprehensive system overviews are provided by Medtronic staff to enhance understanding.

An integral component of this stage is case observation, where surgeons and decision-makers visit an established robotic epicenter to witness the Hugo™ RAS platform in live clinical use. These observations can be organized as standalone 1-day events or combined with a “Hugo™ RAS Discovery Course” at a training center the day prior, complemented by an evening educational presentation.

#### Hugo™ RAS hernia discovery case observation

Participants will attend a clinical observation day at a designated Hugo™ RAS epicenter. They will have the opportunity to observe live surgical procedures, either directly from within the operating room or remotely via live-streaming to a nearby lecture room.

Between cases, dedicated educational sessions will be provided by the epicenter’s faculty, covering both technical and procedural aspects of the Hugo™ RAS platform.

Eligible participants include surgeons interested in robotic surgery who do not yet have access to the Hugo™ RAS system, hospital administrators, and heads of surgical departments evaluating the acquisition of a robotic platform.

#### Hugo™ RAS hernia discovery course

Participants will first attend a Hugo™ RAS Discovery Course at the ORSI Academy Training Center. During this course, they will receive a comprehensive introduction to the Hugo™ RAS robotic system, including an overview of the platform’s technical specifications, the opportunity to inspect the hardware, and hands-on experience with the robotic console through simulator-based exercises.

### Stage II—technology training

1. **Technology training in trainee’s hospital**
  This stage represents the preclinical preparation phase. Once the installation or planned acquisition of a Hugo™ RAS system is confirmed and a general surgeon intends to initiate its clinical use, a structured training pathway with clear timelines becomes essential.
  Comprehensive preparation of the entire surgical team—including the surgeon, first assistant, and nursing staff—is critical for safe and efficient system integration. To optimize the time spent at the ORSI Academy, initial education should be completed at the trainee’s hospital or through online modules and structured assessments.
  The first step is ensuring the team is familiar with the Hugo™ RAS system’s hardware, terminology, and key components. Before attending the ASCEND training at ORSI Academy, the primary surgeon must complete the simulator-based curriculum, consisting of 17 Mimic simulator exercises, each repeated 5 times to achieve documented proficiency. This certification is a prerequisite for participation in the on-site training.
  Technology-specific instruction at the trainee’s hospital, led by a Medtronic Start-up Specialist, covers both the surgical team and operating room staff, ensuring adequate preparation before progressing to the training center.
  This preparatory process reduces the duration of on-site training from 4 to 3 days. The structure and content of the 3-day program will be detailed in the following section.
2. **Technology training in training center**
***Day 1—training center activities***
  The first day of the program takes place at the ORSI Academy Training Center and is delivered by Medtronic staff, with additional support from an ORSI fellow during the afternoon session.
***Morning session***
  The morning is dedicated to consolidating and assessing the knowledge and skills previously acquired during hospital-based training. The activities include:
  Review and reinforcement of the technical training provided at the trainee’s home institution
  Formal evaluation of the surgeon’s simulator-based skills
  Team-based practical exercises, focusing on the preparation and docking of the Hugo™ RAS robotic system
  Essential emergency response training, ensuring all team members are familiar with managing potential intraoperative system issues
***Afternoon session***
  The afternoon session focuses on advanced hands-on training for both the surgical and nursing teams:
  Additional docking practice for the first assistant and nursing staff
  Surgeon-focused console training, including two repetitions of the basic skills task including chicken anastomosis exercise and two repetitions of the chicken leg dissection task [5]. These exercises can be objectively assessed using the Proficiency-Based Progression (PBP) metrics to ensure skill acquisition meets the required standard (Fig. 1) [6].

### Stage IIIA—skills acquisition with tissue handling porcine model

***Day 2—training center activities***

The second day of the program, delivered by Medtronic staff in collaboration with an ORSI fellow, focuses primarily on tissue handling and procedural simulation.

Using a high-fidelity porcine anesthetized model, the training replicates real-life surgical conditions to enhance practical skills acquisition. This environment allows the surgical team to practice procedural workflows, apply the set-up guides, perform robotic docking, and execute safe and efficient instrument exchanges.

**Morning session—robot-assisted cholecystectomy**

The morning is dedicated to performing a robot-assisted cholecystectomy on the porcine model. This session includes:

- Preparation and draping of the Hugo™ RAS system
- Docking of the robotic platform, specifically adapted to the requirements of cholecystectomy
- Instrument set-up and system configuration using the standardized set-up guides

**Fig. 2:**
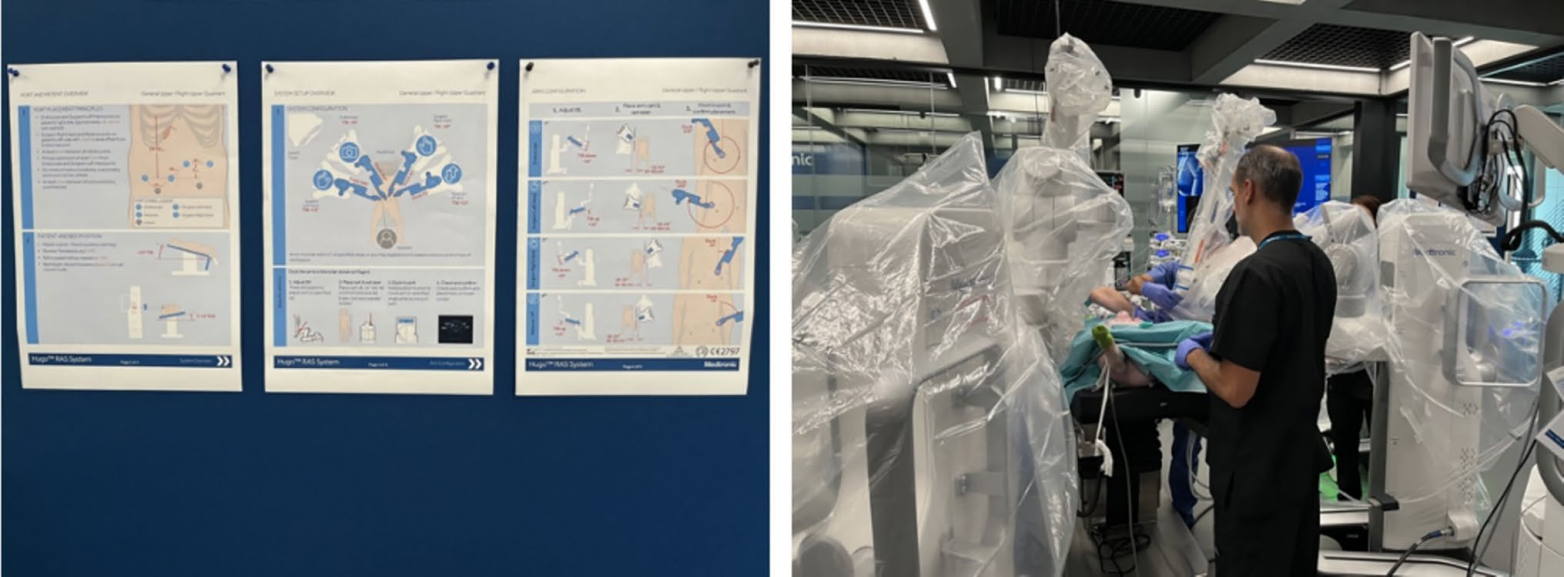
Image showing a training session on the Hugo™ RAS system, performing a robotic cholecystectomy strictly following the procedural workflow

The cholecystectomy procedure is selected due to its relatively low anatomical complexity, allowing the primary focus to be placed on mastering robotic docking techniques, instrument handling, and adherence to procedural workflows, rather than on surgical complexity itself (Fig. 2).

**Afternoon session—robot-assisted ventral hernia repair with intra-peritoneal mesh (rIPOM)**

**Fig. 3:**
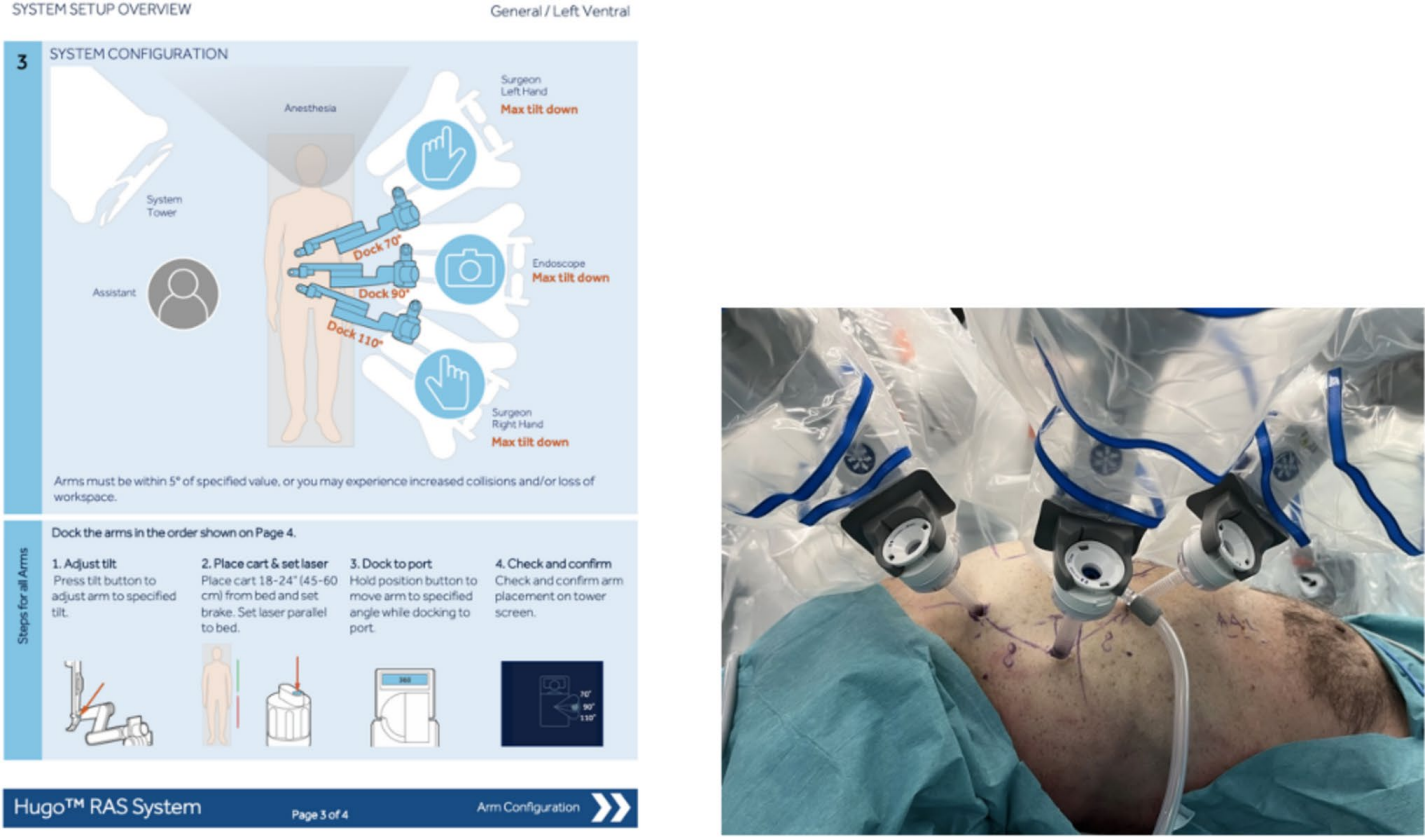
Image showing, on the left, the system set-up overview for robotic ventral hernia repair, and on the right, the placement of three trocars for ventral hernia repair in a porcine model

The afternoon session focuses on performing a robot-assisted ventral hernia repair using an intra-peritoneal onlay mesh (rIPOM) technique on the porcine model (Fig. 3).

The session includes:

- Repetitive practice of system set-up, draping, and docking, following the standardized ventral hernia set-up guide
- Performance of key procedural steps, including fine dissection, suturing, and mesh handling
- Development of camera control skills, as this procedure requires increased camera mobility and repositioning compared to the morning session

Trainee performance will be objectively assessed by the ORSI fellow using validated scoring metrics specific to these training models, ensuring measurable progression towards procedural competence.

### Stage IIIB—skills acquisition with procedural porcine training

***Day 3—training center activities***

The third day focuses on procedural training to prepare the surgeon and team for clinical use of the Hugo™ RAS system. Training is delivered by Medtronic staff and an experienced surgeon-proctor.

The day starts with a session led by the proctor, including a lecture on porcine abdominal wall anatomy and video demonstrations of the SPIRIT and rTARUP simulation exercises, introducing the key procedural steps [7].

This training day follows the structure of the HOT Basic Hernia Courses by ArFiCo Surgical in collaboration with ORSI Academy and ultimately provides the same set of competencies that the European TR300 program by Intuitive Surgical aims to develop.

**Morning session—robot-assisted inguinal hernia repair (SPIRIT model)**

The session begins with a debriefing on the previous day’s activities, including the robot-assisted cholecystectomy and rIPOM procedure. When available, proficiency scores from the ORSI fellow are reviewed, followed by personalized feedback for the trainee.

Next, the SPIRIT model for robotic inguinal hernia repair is performed, following the official set-up guide and standardized procedural steps. This model simulates realistic inguinal anatomy and includes preperitoneal mesh placement to fully cover the myopectineal orifice [7].

The surgeon and team will repeat the SPIRIT model once per groin side to ensure thorough hands-on practice with the Hugo™ RAS system. Performance scoring is recommended to track skill development and support real-time feedback on technical progression.

**Afternoon session—robot-assisted ventral hernia repair (rTARUP model**)

**Fig. 4:**
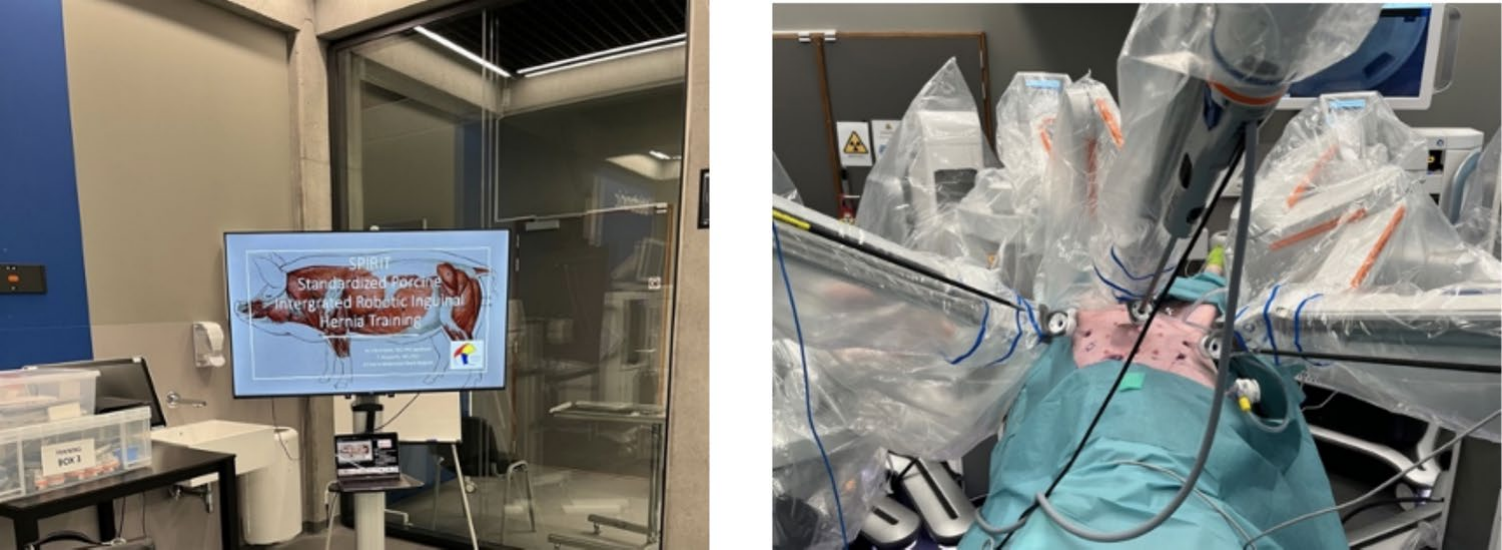
Image showing, on the left, a focus on the porcine model anatomy relevant to robotic abdominal wall surgery, and on the right, trocar placement and robotic docking for robotic TAPP in the porcine model

The afternoon session is dedicated to simulating a robot-assisted ventral hernia repair using the retro-rectus, lateral approach (rTARUP model) (Fig. 4). This session mirrors the complete clinical workflow, including:

- Application of the standardized set-up guide
- Robotic system preparation and docking
- Execution of the retro-rectus hernia repair

This advanced simulation provides the team with additional exposure to complex procedural steps and reinforces familiarity with system handling, ensuring optimal preparation for subsequent clinical implementation.

## Debriefing and conclusion

At the end of the 3-day program, a structured debriefing is held to review the trainee’s performance, provide feedback, and evaluate the overall effectiveness of the training. This session reinforces key learning points and identifies areas for further improvement.

All participants, including bedside assistants, first assistants, and console surgeons, receive official certification upon successful completion of the course, recognizing their achievement of the required standards.

A final discussion between the trainee surgeon and the proctor focuses on planning the clinical adoption of the Hugo™ RAS system. This ensures that all technical, institutional, and logistical requirements are addressed, supporting a safe and confident transition to routine clinical practice.

### Stage IV—first in human

#### Recommendations for initiating clinical practice

To ensure a safe and effective transition to clinical use of the Hugo™ RAS system, surgeons are advised to begin with low-complexity hernia cases. Robot-assisted inguinal hernia repair is considered the ideal starting procedure due to its standardized technique and manageable learning curve.

The first clinical cases should take place within 14 days of completing the training to maintain familiarity with the system and reinforce technical skills. Scheduling at least one robotic hernia procedure per week during the first 2 months is recommended to support continuous practice and skill development.

**Fig. 5:**
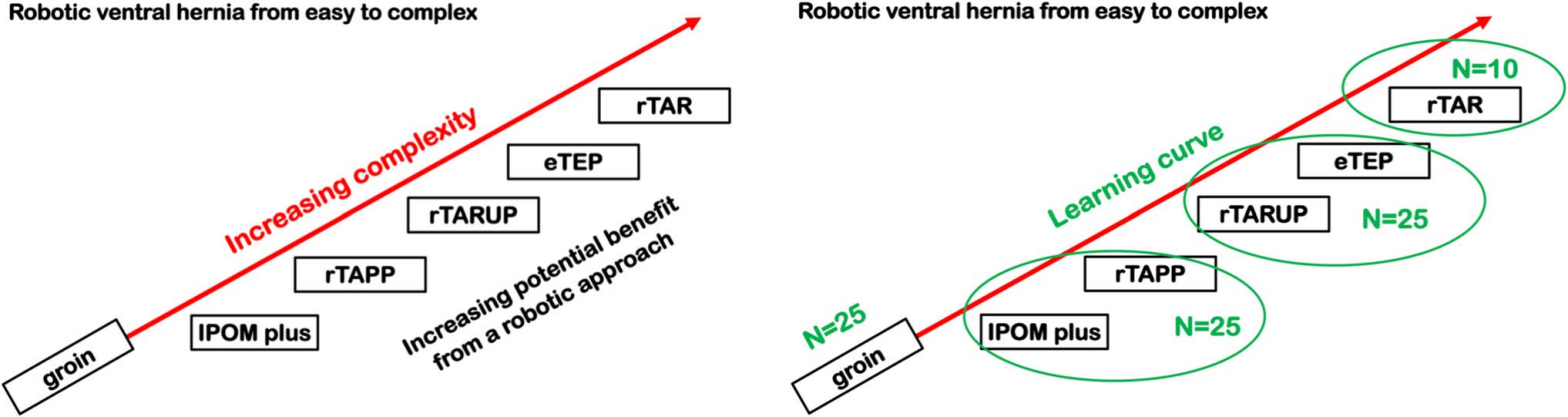
Image depicting the training pathway recommended for surgeons approaching robotic abdominal wall surgery [4]. Figure reproduced from Vierstraete et al. [4], under the terms of the Creative Commons Attribution (CC BY) license

Whenever possible, the same surgeon-proctor from Day 3 of the training should be present for the first human cases. This continuity offers personalized guidance and facilitates a smooth, confident integration of the Hugo™ RAS system into clinical practice (Fig. 5).

### Stage V—clinical confidence building

A structured, stepwise approach to case selection is recommended, following the adoption model described by Vierstraete et al. [4]. This method starts with low-complexity procedures and gradually progresses to more technically demanding surgeries.

Such a progressive learning curve allows surgeons to build proficiency and confidence with the Hugo™ RAS system while minimizing risks and prioritizing patient safety. This strategy supports both optimal skill acquisition and consistent, successful outcomes during the early stages of robotic program implementation.

### Stage VI—advanced skills with procedural porcine training

This advanced training session follows the structure of the HOT Advanced Hernia Courses, organized by ArFiCo Surgical in collaboration with the ORSI Academy, and adopted by Intuitive Surgical as part of their European TR400 program. The model has been successfully tested during an advisory board session focused on robotic hernia surgery with the Hugo™ RAS system.

After completing approximately 50–75 low-complexity robotic hernia repairs, surgeons are encouraged to progress to more complex procedures. To support this transition, we developed a high-fidelity anesthetized porcine model for practicing posterior component separation, also known as robotic transversus abdominis release (roboTAR).

This model is based on an in-depth anatomical study of the porcine abdominal wall, the results of which are presented during the HOT Advanced Hernia Course. Its anatomical accuracy provides a realistic, effective training environment to prepare surgeons for the clinical adoption of the TAR technique [8].

**Fig. 6:**
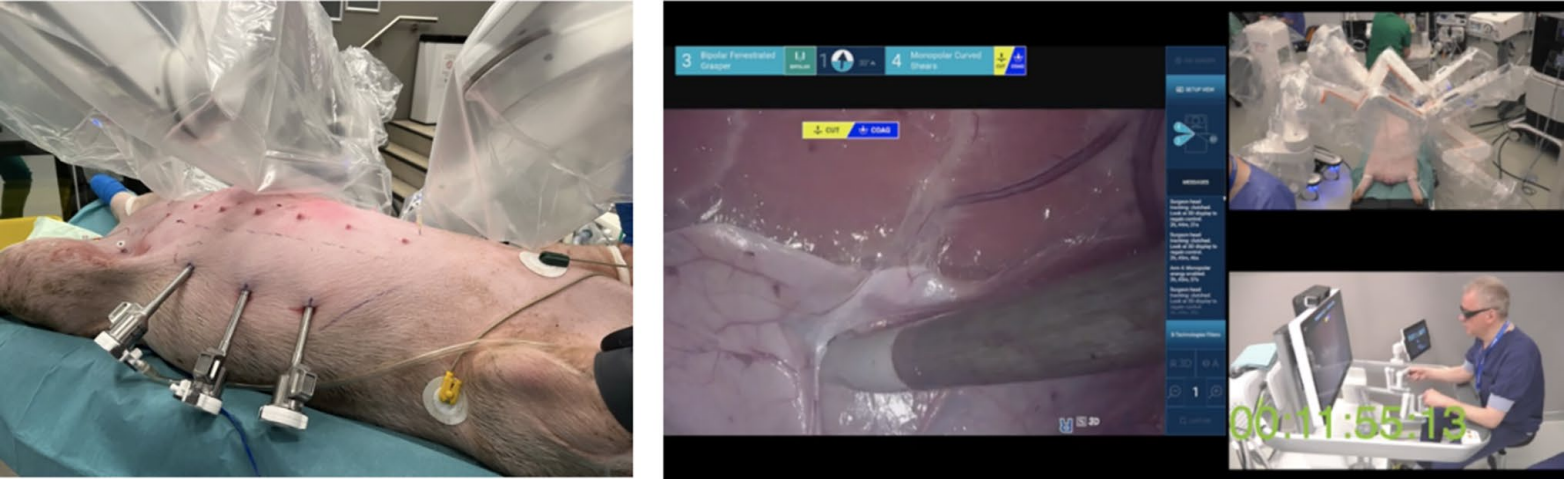
Image showing the roboTAR procedure on a porcine model using the Hugo™ RAS system, depicted from multiple perspectives

To date, several advanced hernia courses have been conducted with both the Hugo™ RAS and da Vinci platforms. Surgeon feedback has been highly positive, particularly regarding the model’s anatomical fidelity and its role in supporting safe and confident integration of TAR into clinical practice (Fig. 6).

### Stage VII—proctor skills development

As robotic-assisted hernia surgery continues to expand, the demand for qualified proctors to mentor new surgeons is expected to grow. To ensure consistent, high-quality training, we recommend implementing structured “Train the Trainer” programs.

These sessions provide proctors with in-depth knowledge of the ASCEND pathway and promote standardized education across centers. Upon completion, proctors receive additional certification specific to the use of the Hugo™ RAS system for hernia surgery.

This certification ensures that proctors can confidently and effectively guide future surgeons, supporting the safe and consistent clinical integration of robotic-assisted hernia repair.

## Discussion

The development of structured training programs for robotic-assisted surgery represents a key advancement in surgical education, especially as systems like the Hugo™ RAS platform continue to expand across Europe and internationally. The Hernia ASCEND training pathway integrates technical, anatomical, and procedural education while ensuring that both individual surgeons and surgical teams acquire the necessary skills for safe clinical practice. The structured format draws on established models, such as the da Vinci training framework, but is adapted to the specific ergonomics, workflows, and system architecture of the Hugo™ RAS platform.

The use of PBP and modular training allows for stepwise skill acquisition, with clear performance benchmarks ensuring that trainees advance only when ready [5, 6]. Early experiences suggest that this approach improves surgeon confidence and facilitates smoother clinical integration [9–11]. The incorporation of virtual simulation, objective performance metrics, and team-based education further enhances the training’s effectiveness, preparing not only the console surgeon but also assistants and nursing staff for real-world surgical scenarios [12–16].

Robotic surgery inherently requires coordinated team performance, and growing evidence indicates that structured interdisciplinary training improves both technical outcomes and non-technical skills such as communication and crisis management [16, 17]. Digital tools, including cloud-based video platforms, further support continuous learning, remote assessment, and training standardization across institutions.

Despite these advancements, variability in credentialing criteria persists [13, 18, 19]. Current practices often rely on case volume thresholds, which may not correlate with actual proficiency. There is a growing consensus that training programs should incorporate validated assessment tools, objective performance criteria, and formal proctoring to ensure safe adoption.

The increasing demand for robotic platforms, coupled with their complexity, highlights the need for scalable, standardized training models. Regional centers, remote learning, and collaborative programs can help overcome logistical barriers, but success depends on institutional commitment and ongoing curriculum refinement.

Looking forward, large-scale multicenter studies will be required to validate the ASCEND pathway by correlating training outcomes with clinical performance, particularly patient safety and complication rates, while emerging technologies such as artificial intelligence, augmented reality, and haptic feedback are expected to further personalize and enhance training experiences [20].

While industry support is indispensable in providing access to platforms, simulators, and technical expertise, the responsibility for certification, benchmarking, and quality control should rest with independent scientific societies such as the EHS, thereby ensuring neutrality, patient safety, and international comparability.

In conclusion, structured training pathways for the Hugo™ RAS system, grounded in evidence-based principles and supported by modern technologies, provide a robust foundation for the safe and effective expansion of robotic hernia surgery. Continued validation and adaptation of these programs will be essential to meet the growing demands of robotic-assisted surgery while maintaining high standards of patient care and surgical excellence.

## Conclusion

The Hernia ASCEND Hugo™ RAS Training Pathway provides a comprehensive, standardized framework for the safe and effective adoption of robotic-assisted abdominal wall surgery. Through its structured progression, and emphasis on hands-on proficiency, ASCEND fosters surgical excellence, facilitates safe clinical introduction, and promotes scalable expertise across healthcare systems.

## Supplementary Information

Below is the link to the electronic supplementary material.

Supplementary file1 (DOCX 173 KB)

## Data Availability

No datasets were generated or analyzed during the current study.
