# Supplementary material for: Proposal for the Hernia ASCEND Hugo™ RAS training pathway: acquisition of skills by comprehensive exercise-based nimbleness and dexterity training
Source: J Robot Surg. 2025 Aug 26;19(1):516. doi: 10.1007/s11701-025-02704-8 (PMC12380880; doi:10.1007/s11701-025-02704-8)
Supplement: Supplementary file 1 — Supplementary file1 (DOCX 173 KB) [file 11701_2025_2704_MOESM1_ESM.docx]

# Supplementary materials

# Figure 1s

#
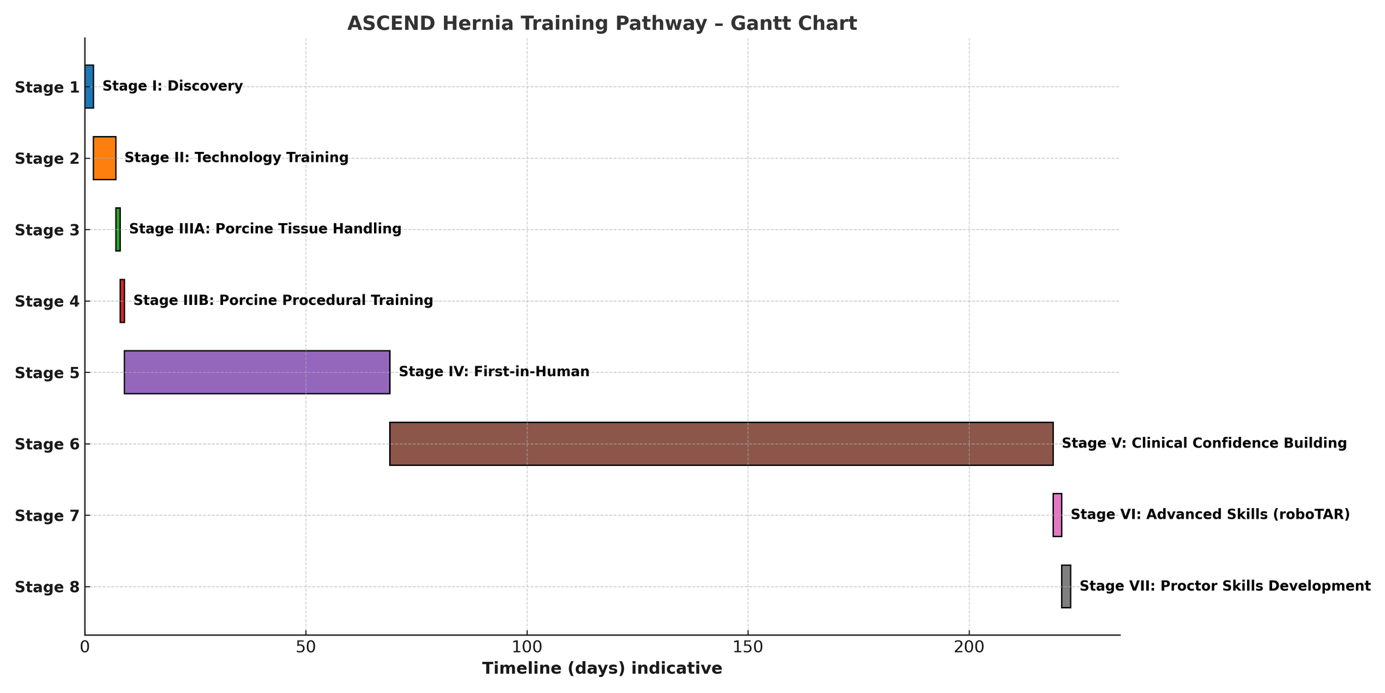


# Table 1s. Draft Table

Stages of the Hernia ASCEND Hugo™ RAS Training Pathway

| Stage | Setting | Target Learners | Learning Objectives | Assessment Tools | Progression Gates |
| --- | --- | --- | --- | --- | --- |
| I. Discovery | Congresses, demo truck, epicenter observation | Surgeons, hospital administrators, heads of dept. | Familiarization with Hugo™ RAS platform, hardware exposure, observation of live cases | Case observation feedback, course participation | Completion of introductory exposure and observation |
| II. Technology Training | Trainee’s hospital + ORSI Academy training center | Surgeon, first assistant, OR staff | Technical system knowledge, simulator proficiency, team docking, emergency response | Simulator curriculum (17 tasks ×5 reps), docking checklists, PBP metrics | Simulator certification and team readiness documented |
| IIIa. Skills Acquisition (Porcine – Tissue Handling) | Training center (anesthetized porcine model) | Surgeon, assistants, nursing staff | Docking, tissue handling, basic dissection, suturing, rIPOM technique | ORSI fellow scoring, validated porcine model checklists | Achieving defined proficiency scores on cholecystectomy and rIPOM tasks |
| IIIb. Skills Acquisition (Porcine – Procedural) | Training center (porcine models: SPIRIT, rTARUP) | Surgeon + team | Reproduce procedural workflows in inguinal and ventral hernia models | Validated checklists, proctor evaluation | Certification of competency in SPIRIT and rTARUP |
| IV. First in Human | Clinical OR with proctor supervision | Surgeon + team | Safe introduction to clinical practice (low-complexity inguinal hernia) | Proctor feedback, case log review | At least one supervised case/week in first 2 months |
| V. Clinical Confidence Building | Clinical OR | Surgeon + team | Stepwise progression from low- to high-complexity hernia cases | Case volume tracking, outcome monitoring | 50–75 successful low-complexity cases completed |
| VI. Advanced Skills (Porcine – roboTAR) | Training center (advanced porcine model) | Experienced robotic hernia surgeons | Posterior component separation (roboTAR) | Advanced porcine model validation, faculty scoring | Successful completion of advanced course requirements |
| VII. Proctor Development | Training center | Experienced surgeons | Train-the-trainer, proctor certification | Standardized proctor certification pathway | Recognition as certified Hugo™ RAS hernia proctor |

# Supplementary Table S2. Assessment tools and proficiency criteria across ASCEND stages

| Stage | Assessment Tools | Proficiency Criteria / Outcome | Validation Status |
| --- | --- | --- | --- |
| Simulator training | Mimic™ simulator curriculum (17 tasks × 5 reps) with PBP metrics | ≥80% task completion within benchmark time; embedded pass/fail cut-offs | Validated |
| Porcine hernia models (SPIRIT, rTARUP, roboTAR) | Structured metrics scoring common and critical errors | Binary outcome: Proficient / Not proficient | Delphi consensus under development |
| Clinical training phase | Structured logbook, proctor evaluation | Safe progression documented; proctor sign-off required | Adapted from validated global rating scales |
